# Transcriptome and Genome Analyses Applied to Aquaculture Research

**DOI:** 10.3390/biology11091312

**Published:** 2022-09-04

**Authors:** Patricia Pereiro

**Affiliations:** Institute of Marine Research (IIM), National Research Council (CSIC), Eduardo Cabello, 6, 36208 Vigo, Spain; patriciapereiro@iim.csic.es; Tel.: +34-986231930

The Special Issue “Transcriptome and Genome Analyses Applied to Aquaculture Research” had great success among the researchers specialized in different fields of aquaculture. It reached 27 published manuscripts covering different interests (immunology, response to stressors, sexual dimorphism, development, etc.) in a variety of fish and shellfish, and even in turtles. Different transcriptome (mRNAs and non-coding RNAs –ncRNAs–), genome (Single Nucleotide Polymorphisms –SNPs–), and metatranscriptome analyses were conducted to unravel those different aspects of interest.

Understanding the immune response to different pathogens is pivotal for developing breeding selection strategies to improve the resistance to diseases or for formulating new treatments, among other objectives. In this sense, massive analyses of the immune response have attracted a lot of interest and are being conducted in different species of interest for the aquaculture industry.

Parasites, and especially endoparasites, are highly problematic for fish production due to the absence of treatments and vaccines on the market for most of them. Because of this, the study of the response to these pathogens could provide interesting clues aimed to improve the fish’s resistance to endoparasites. Two works included in this Special Issue analyzed the response of the flatfish turbot (*Scophthalmus maximus*), a very valuable fish species both in Europe and China, to two different endoparasites causing important economic losses. Whereas Valle et al. [1] explored the transcriptome response of the turbot peritoneal cells at 1, 2, and 4 h post-infection (hpi) with the ciliate parasite *Philasterides dicentrarchi*, as well as the transcriptome of the own parasite after the challenge, Ronza et al. [2] carried out transcriptomic and histopathological analyses of the thymus after infection with the myxozoan parasite *Enteromyxum scophthalmi*.

But several fish viruses are also problematic for the same reasons: no vaccines nor therapeutic treatments commercially available. Different components of the antiviral response were analyzed in this Special Issue. Tripartite motif proteins (TRIMs) are increasingly well known for their antiviral immune functions in mammals, although their exact function in fish still needs to be fully elucidated, which is hindered by the massive gene expansion of the teleost TRIM family. Qin et al. [3] identified 42 *trim* genes in the grass carp (*Ctenopharyngodon idella*) genome and conducted different domain and phylogenetic analyses to classify them. The authors also found that 11 of these TRIMs were significantly induced during grass carp reovirus (GCRV) infection and, by co-expression analyses, they identified three *trim* genes that could be considered as a part of the type I interferon response [3]. On the other hand, Bello-Perez et al. [4] analyzed the expression of the seven C-reactive proteins (CRPs) previously identified in zebrafish (*Danio rerio*) under basal conditions and after spring viraemia of carp virus (SVCV) infection in different tissues. Most of these *crp* genes were altered after the viral challenge in different tissues, including the skin. This tissue also showed a higher expression of these genes in the recombinant activation gene 1 (*rag1*) mutant zebrafish compared to their wild-type counterparts, suggesting an overcompensation of the innate immune in the absence of the adaptive one [4]. Finally, Pereiro et al. [5] analyzed by RNA-Seq the repertoire of long non-coding RNAs (lncRNAs) in the brain and head kidney from European sea bass (*Dicentrarchus labrax*) and identified those lncRNAs significantly modulated after infection with nodavirus. By identifying the neighboring coding genes of these differentially expressed lncRNAs, the potential functions affected by these lncRNAs were determined [5].

But, in addition to the study of the immune response to different pathogenic challenges, the knowledge of the immunological properties of the different immune cell types is also a cornerstone for fish immunologists. Perdiguero et al. [6] conducted single-cell RNA sequencing to analyze for the first time in teleost the transcriptional profile of single B cells from rainbow trout (*Oncorhynchus mykiss*) peripheral blood. Based on their transcriptome profiles, the authors identified ten different B cell subpopulations. This information provides valuable information to understand the biology of teleost B cells and could serve as a source of potential markers to be used to differentiate B cell subsets [6].

In addition to fish, several pathogens also impact the production of other economically relevant organisms produced in aquaculture systems. This is the case of the Chinese soft-shelled turtle (*Trionyx sinensis* or *Pelodiscus sinensis*), an important cultured reptile in East Asia taken as a food resource, especially in China and Japan. Lv et al. [7] conducted RNA-Seq analyses of liver and spleen from uninfected turtles and two groups of turtles with different susceptibility to *Aeromonas hydrophila* infection. Whereas the diseased turtles showed a high induction of genes encoding for pro-inflammatory cytokines, pattern recognition receptors (PRRs), and apoptosis-related molecules and low phagocytic response, asymptomatic turtles showed the opposite pattern. All these data are very useful to understand the molecular basis of the resistance to the hemorrhagic sepsis caused by *A. hydrophila* in Chinese soft-shelled turtles [7]. On the other hand, hepatopancreas necrosis disease of the Chinese mitten crab (*Eriocheir sinensis*) is causing huge economic losses in China, although the pathogens potentially involved in this disease have not yet been identified. To elucidate this question, Shen et al. [8] conducted metatranscriptome analyses of the hepatopancreatic flora of diseased crabs with mild symptoms, diseased crabs with severe symptoms, and crabs without visible symptoms. The authors observed a decrease in the prevalence of the bacteria *Absidia glauca* and *Candidatus Synechococcus spongiarum*, whereas the prevalence of *Spiroplasma eriocheiris* increased in the hepatopancreatic flora of diseased crabs. Moreover, the flora from diseased crabs showed a higher expression of genes involved in certain functions that could be also involved in the pathological mechanism [8].

Chronic or acute inflammation is an undesirable condition in fish aquaculture, which could be a consequence of inadequate aquaculture rearing conditions (husbandry, transportation, crowding densities, water parameters, etc.) or infections. These stressful environments can compromise fish growth, welfare, and immunity. The neuroendocrine-immune axis is a bi-directional network; for instance, the immune response conditions the stress response and vice versa. It is known that opioid receptors are involved in regulatory mechanisms of both the immune and the stress responses in mammals. Nevertheless, their exact roles and responsiveness to immune stimulation in the brain are still not known, especially in non-mammalian species. To better understand the link between the peripheral immune signaling and the central neuroendocrine responses, Azeredo et al. [9] and Peixoto et al. [10] analyzed the effect of acute and chronic inflammation, respectively, induced by the intraperitoneal injection of Freund’s Incomplete Adjuvant in European sea bass. Azeredo et al. observed that different neuroendocrine and opioid receptors were altered during acute inflammation in different brain regions, which demonstrates the interconnection between peritoneal inflammation and brain neuroendocrine response, as well the existence of potential site-specific functions for these receptors in the different regions of the brain [9]. On the other hand, Peixoto et al. analyzed different immune parameters after chronic inflammation, which was characterized by higher leukocyte numbers in the blood accompanied by an intensification of the respiratory burst and higher transcription levels of *cxcr4* and *il34* in the head kidney [10]. Nevertheless, contrary to that observed by Azeredo et al. [9] in the European sea bass brain, opioid receptors seem not to be affected by inflammation in head kidney cells [10].

Stress can significantly impact different physiological aspects of the animals, which could result in a low growth rate, higher susceptibility to diseases, and even a low meat quality. There are a variety of stressors that can induce a pernicious effect in the aquaculture species (changes in temperature, pH, salinity, water oxygen saturation, handling, etc.) The impact of different stressors was analyzed in different works included in this Special Issue. Dettleff et al. [11] evaluated the effects of high-temperature stress on the liver of red cusk-eel (*Genypterus chilensis*) through different approaches, including RNA-Seq analysis. The results revealed that this stressor induced oxidative damage in the liver and affected the expression of a multitude of genes, which are mainly involved in the unfolded protein response, heat shock response, and oxidative stress, among others, providing a rich source of information to be considered for the aquaculture and fisheries industry of this species under a climate change scenario [11]. Interestingly, Naya-Catalá et al. [12] found that gilthead sea bream (*Sparus aurata*) juveniles acclimated for 45 days to mild-hypoxia and then returned to normoxia showed an increased contribution of lipid metabolism to the whole energy supply to preserve the aerobic energy production, a better swimming performance regardless of changes in feed intake, as well as reduced protein turnover and improved anaerobic fitness with the restoration of normoxia. These results indicate that mild-hypoxia conditioning could serve as a promising prophylactic measure to prepare these fish for predictable stressful events [12].

Salinity alteration is also a very frequent stressor that aquatic animals need to cope with. Gills are one of the most important tissues in osmoregulation and therefore, to known the mechanisms affected in this tissue could help to better understand the adaptation to salinity. Malik and Kim [13] conducted a meta-analysis of publicly available RNA-Seq datasets to identify differentially expressed genes in the Chinese mitten crab gills under different salinity conditions. The authors found a great modulation of many different types of ion transporters and channels (transportome) under different salinity conditions that may serve as biomarkers for osmoregulation. On the other hand, Li et al. [14] conducted a systematic analysis of 95 heat shock protein (*hsp*) genes in spotted sea bass (*Lateolabrax maculatus*) gills, a fish species that can widely adapt to diverse salinities from fresh to seawater, and moderately adapt to high alkaline water. The study revealed that these genes were mainly affected in the gills by alkalinity stress, responding with a coordinated modulation of *hsp*40-70-90 families [14]. But changes in salinity are also a part of the life cycle of the diadromous (catadromous and anadromous) fish species. Smoltification is a complex physiological process that prepares freshwater fish for successful osmoregulation in seawater, and it has been mainly studied in salmonid species. Nevertheless, how non-coding RNAs can contribute to these complex physiological alterations remained practically unexplored. Whereas Valenzuela-Muñoz et al. [15] analyzed the mRNA and miRNA profiles in gills, intestine, and head kidney from Atlantic salmon (*Salmo salar*) exposed to a gradual salinity change or to a salinity shock, Shwe et al. [16] studied their modulation in the liver during smoltification. The results revealed a set of miRNAs associated with smoltification and allowed us to predict their highly confident target genes [15,16]. Additionally, Valenzuela-Muñoz et al. [15] also found that salinity changes induced a broad modulation of transposable elements, which suggests a relevant role of these molecules in the smoltification process.

Gender dimorphism has drawn a lot of attention due to the different growth rates and body sizes of males and females depending on the species. This is the case of the Chinese soft-shelled turtles, with males exhibiting the best growth pattern. Consequently, to understand the sex determination- and differentiation-related genes could help to easily achieve single-sex breeding populations. In this sense, Zhu et al. [17] carried out transcriptome analyses of the ovaries and testes of these turtles using RNA-Seq to identify the genes, lncRNAs, miRNAs, and circRNAs that could be involved in gender dimorphism and well as to determine the relationship between genes and ncRNAs in the regulatory mechanism of sex differentiation. Pseudo-female turtles, which have a female phenotype and male genotype after estradiol administration, can be used to obtain an all-male offspring. Nevertheless, the genes involved in the gonad differentiation and sex reversal of Chinese soft-shelled turtles were poorly known. Wang et al. [18], by analyzing the transcriptome profile of male, female and pseudo-female gonads found that differences between males and pseudo-females were mainly related to steroid hormone synthesis at the transcriptome level. Moreover, the SRY-related high-mobility group (HMG)-box (SOX) family of genes seems to be playing an important role in the process of sex reversal from male to pseudo-female, being *sox3* induced by exogenous estrogen and *sox8* and *sox9* inhibited [18]. But, in addition to gender, growth dimorphism could be also observed in tetraploid individuals compared to their diploid wild-type counterparts. Because of this, Luo et al. [19] compared the transcriptome profiles through RNA-Seq of different tissues (brain, gonad, liver, and muscle) from diploids and tetraploids of both sexes of the freshwater fish loach (*Misgurnus anguillicaudatus*). Their results suggested that energy metabolism levels, steroid hormone synthesis, and fatty acid degradation abilities might be important reasons for the sexual and polyploidy dimorphisms in loaches, three processes that were clearly differentiated between fast-growing loaches (tetraploids, females) and slow-growing loaches (diploids, males) [19].

For several fish species, interspecific hybrids also show higher feed conversion, disease resistance, carcass yield, and harvestability compared to both parental species, which is known as heterosis or hybrid vigor. This is the case of the hybrid between female channel catfish (*Ictalurus punctatus*) and male blue catfish (*Ictalurus furcatus*). Interestingly, heterosis for these hybrids only occurs in small culture units (pond culture) but not in tank culture, being therefore environment-dependent. Wang et al. [20] conducted different morphometric, immune, and metabolic measurements and analyzed through RNA-Seq the differences in the liver transcriptome among channel catfish, blue catfish, and their reciprocal F1s reared in tanks. The results showed that the tank environment resulted in a higher growth performance for channel catfish, which could be related to a lower immune function and a higher expression of genes involved in fatty acid metabolism/transport [20]. On the other hand, hybrids showed a higher level of blood glucose, which can be explained by the phenomenon known as transgressive expression (overexpression/underexpression in F1s than the parental species); indeed, a total of 1140 transgressive genes were identified in F1 hybrids, showing an enrichment in glycan degradation function [20]. This gene expression and physiological differences could be contributing to the lack of heterosis in the tank culture environment.

Polyunsaturated fatty acids (PUFAs) content is a desirable trait to be selected in fish aquaculture. Zhang et al. [21] identified single nucleotide polymorphisms (SNPs) in the coding regions of the fatty acid elongase 5 genes, *elovl5a* and *elovl5b,* in common carp (*Cyprinus carpio*) and correlated them with the PUFAs content. Three SNPs, one in *elovl5a* and two in *elovl5b*, were found to be associated with higher PUFA levels, and could be used in the future as markers for selective breeding in common carp [21]. But, in addition, to improve the PUFAs content in this way, optimal fish nutrition can also significantly impact the lipid composition, among others aspects. Katan et al. [22] used microarrays to examine transcriptome differences in the liver of Atlantic salmon fed with a diet containing a high ratio of omega-6 to omega-3 (ω6:ω3) fatty acids and those fed with a low ω6:ω3 ratio. The results revealed differences in the expression of genes involved in the immune response, lipid metabolism, cell proliferation, and translation, and determined that two helicases with zinc finger 2 (*helz2*) paralogues were positively correlated with ω3 and negatively with ω6 fatty acids both in liver and muscle, indicating their potential as biomarkers of tissue ω6:ω3 variation [22].

Fish development has been extensively studied since it could help to improve different production bottlenecks, such as massive mortality episodes or malformations. Flatfishes have the particularity that undergoes a dramatic metamorphosis to change their body pattern from a pelagic bilateral symmetric larva to a benthonic flat asymmetric juvenile. Since this process is mainly controlled by the brain, Guerrero-Peña et al. [23] conducted RNA-Seq analyses of the brain from different developmental stages of turbot (pre-metamorphic, metamorphic climax, and post-metamorphic) to understand the transcriptome modulations occurring during the metamorphosis. The authors found 1570 genes differentially expressed in the three developmental stages, being the climax stage of metamorphosis characterized by a high expression of genes involved in inflammation and apoptosis, which could be related to processes of larval tissue inflammation, resorption, and replacement, as occurs in other vertebrates [23]. Moreover, their results confirmed the relevance of the thyroid stimulating hormone in the induction of the teleost metamorphosis process [23].

But not only does the expression pattern of the protein-coding genes changes during development, the expression of ncRNAs, as regulators of the gene expression, is also fine-tuned during development. To obtain the complete miRNAome of lumpfish (*Cyclopterus lumpus*), Chakraborty et al. [24] conducted miRNA analysis of different organs from adult individuals and entire embryos and larvae. They obtained 443 unique mature miRNAs from 391 conserved and eight novel miRNA genes, which represent an essential reference for future expression analyses. Additionally, the expression patterns of specific conserved miRNAs in particular tissues and developmental stages indicated that they are involved in organ- and developmental stage-specific functions reported in other teleost [24]. To obtain as most complete as possible a first transcriptome of edible red sea urchin, *Loxechinus albus* was the objective of Antiqueo et al. [25]. For this, the authors sequenced gonads, intestines, and coelomocytes of juvenile urchins. After de novo assembly, a total of 185,239 transcripts were obtained, of which 57,106 (31%) were successfully annotated. Comparative transcriptome analyses revealed that differentially expressed transcripts among tissues were specialized in different biological processes according to the specific functions of these tissues [25].

Finally, this Special Issue also included research about the development of the intermuscular bones (IBs), which negatively affect the edibleness and economic value of fish. Comparing species with and without epineural and epipleural IBs or fish strains with different abundances of them could provide interesting clues for reducing the number of IBs. Zhou et al. [26] analyzed different stages of myosepta development using morphological and transcriptomics analyses in blunt snout bream (*Megalobrama amblycephala*) and Nile tilapia (*Oreochromis niloticus*), fish species with and without epineural and epipleural IBs, respectively. Among other results, the authors identified a repertoire of genes that may play important roles in IB development, with could provide interesting information for further studies [26]. On the other hand, Cui et al. [27] conducted whole-genome resequencing and bulked segregant analysis in three common carp strains with a different abundance of IBs to identify SNPs that could be associated with the number of IBs. The 21 SNPs significantly associated with the abundance of IBs could serve as potential markers for selective breeding to generate IB-reduced common carps [27].
